# Saliva Neurofilament Light Chain Is Not a Diagnostic Biomarker for Neurodegeneration in a Mixed Memory Clinic Population

**DOI:** 10.3389/fnagi.2021.659898

**Published:** 2021-05-10

**Authors:** Helena Sophia Gleerup, Federica Sanna, Peter Høgh, Joel Simrén, Kaj Blennow, Henrik Zetterberg, Steen Gregers Hasselbalch, Nicholas J. Ashton, Anja Hviid Simonsen

**Affiliations:** ^1^Department of Neurology, Danish Dementia Research Centre, Copenhagen University Hospital, Copenhagen, Denmark; ^2^Institute of Neuroscience and Physiology, Department of Psychiatry and Neurochemistry, The Sahlgrenska Academy at the University of Gothenburg, Mölndal, Sweden; ^3^Regional Dementia Research Centre, Department of Neurology, Zealand University Hospital, Roskilde, Denmark; ^4^Department of Clinical Medicine, Faculty of Health and Medical Science, University of Copenhagen, Copenhagen, Denmark; ^5^Clinical Neurochemistry Laboratory, Sahlgrenska University Hospital, Mölndal, Sweden; ^6^Department of Neurodegenerative Disease, UCL Institute of Neurology, London, United Kingdom; ^7^UK Dementia Research Institute at UCL, London, United Kingdom; ^8^Wallenberg Centre for Molecular and Translational Medicine, University of Gothenburg, Gothenburg, Sweden; ^9^King’s College London, Institute of Psychiatry, Psychology and Neuroscience, Maurice Wohl Institute Clinical Neuroscience Institute, London, United Kingdom; ^10^NIHR Biomedical Research Centre for Mental Health and Biomedical Research Unit for Dementia at South London and Maudsley NHS Foundation, London, United Kingdom

**Keywords:** neurodegeneration, dementia, neurofilament light chain, saliva, plasma, Alzheimer’s disease, biomarker

## Abstract

Neurodegeneration and axonal injury result in an increasing release of neurofilament light chain (NfL) into bodily fluids, including cerebrospinal fluid (CSF) and blood. Numerous studies have shown that NfL levels in CSF and blood are increased in neurodegenerative disorders and monitor neurodegeneration. Saliva is an easily accessible biofluid that could be utilized as a biofluid measurement of Alzheimer’s disease (AD) biomarkers. In this study, for the first time, salivary NfL was measured and compared to plasma NfL in a consecutive cohort of patients referred to cognitive assessments. In two mixed memory clinic cohorts, saliva samples were taken from 152 patients, AD (*n* = 49), mild cognitive impairment (MCI) (*n* = 47), non-AD (*n* = 56), and also 17 healthy controls. In addition, 135 also had a matching plasma sample. All saliva and plasma samples were analyzed for NfL, and the association between saliva and plasma NfL and CSF levels of total tau (t-tau), phosphorylated tau (p-tau), and beta amyloid 1–42 (Aβ42) were investigated. In total, 162/169 had quantifiable levels of salivary NfL by single molecule array (Simoa). No statistically significant differences were found in salivary NfL concentration across the diagnostic groups, but as expected, significant increases were found for plasma NfL in dementia cases (*P* < 0.0001). There was no association between saliva and plasma NfL levels. Furthermore, saliva NfL did not correlate with CSF Aβ42, p-tau, or tau concentrations. In conclusion, NfL is detectable in saliva but does not reflect neurodegeneration in the brain.

## Introduction

Neurodegenerative dementias, specifically Alzheimer’s disease (AD), are an accelerating health and economic issue that affects more than 46.8 million patients worldwide, and it is estimated that, by 2035, this number will likely double without modifiable or preventive treatment (Martin Prince et al., 2015). Cognitive deficits in neurodegenerative dementias are linked to the duration of the disease and are caused by accelerating neurodegenerative processes, among these are neuronal damage and loss. The pathological processes of neurodegenerative dementias begin several decades prior to the clinical expression, and therefore, it is necessary to find new methods to detect these diseases at the preclinical stage to allow for the initiation of future disease-modifying treatments or the inclusion in clinical trials of novel drug candidates (Cummings et al., 2020). Currently, the diagnosis of dementia relies primarily on neurological and psychological assessment, imaging modalities, and analyses of the cerebrospinal fluid (CSF), especially tau, phosphorylated tau (p-tau), and beta amyloid 1–42 (Aβ_42_), some of which are specific to AD pathology. However, some of these methods require special training to perform, are regarded as invasive, and may in a percentage of cases lead to adverse reactions (Costerus et al., 2018). Furthermore, considerable economic resources are often spent, and some imaging methods cause radiation and lack molecular specificity (Lin, 2010). For these reasons, it is essential to develop new non-invasive and inexpensive methods that can differentiate between neurodegenerative and non-neurodegenerative diseases in representative clinical settings. Blood biomarkers have led the way in this respect. Mass spectrometric assays for plasma Aβ_42_ or Aβ ratio have demonstrated high accuracy in detecting cerebral Aβ pathology (Nakamura et al., 2018; Schindler et al., 2019). Blood p-tau is a highly specific pathological blood biomarker for AD pathology and encompasses all diagnostic capabilities of CSF p-tau (Benussi et al., 2020; Karikari et al.,2020a,b; Lantero Rodriguez et al., 2020; Moscoso et al., 2020; O’Connor et al., 2020; Ashton et al., 2021). Nonetheless, while blood biomarkers are considerably less complexed than CSF and molecular imaging, venipuncture is still required to extract the sample that may still limit some populations.

Saliva testing is a potential non-invasive alternative to blood biomarkers. Saliva has already been suggested to be a valid biofluid for biomarker analysis in several areas besides neurology, among these are endocrinological and cardiovascular diseases, cancer, and HIV (Emmons, 1997; Adam et al., 1999; Walt et al., 2007; Sashikumar et al., 2010; Zhang et al., 2010). Furthermore, studies have shown that an altered composition of the proteins and electrolytes in saliva can mirror hormonal, immunological, and metabolic or nutritional changes in the body (Spielmann and Wong, 2011). Increasing evidence points toward saliva being a potential alternative to the current methods used for the analysis of biomarkers for neurodegenerative dementias (Ashton et al., 2019a; Gleerup et al., 2019). A saliva-based test that could differentiate between neurodegenerative and non-neurodegenerative diseases in consecutive mixed memory clinic cohorts not only would be useful in the clinical management of the patients but also could contribute to the effective utilization of clinical resources. The origin of the biomarkers found in saliva is still undefined, but studies have suggested that biomarkers could be excreted directly from the degenerating axons of the parasympathetic cranial nerves that innervate the main salivary glands (Farah et al., 2018). It has also been suggested that the biomarkers are produced or expressed in some or all of the salivary glands (Lee et al., 2017; McGeer et al., 2018) or that the biomarkers are transported from the blood into the saliva by different transport mechanisms depending on the biomarker (Spielmann and Wong, 2011).

Neuronal damage and loss in the central nervous system (CNS) are important steps in the pathophysiology of neurodegenerative dementias, regardless of the primary pathology of the specific dementia diagnosis. Neurofilaments are neuron-specific scaffolding proteins that enable the radial growth of axons (Yuan and Rao, 2017). They are particularly abundant in axons, and small increases of neurofilaments in CSF are observed in an age-dependent manner (Khalil et al., 2020). Neurofilaments are composed of neurofilament light chain (NfL), neurofilament medium chain (NfM), neurofilament heavy chain (NfH), alpha-internexin, and peripherin. NfL and NfH are the most important subunits for the axonal radial growth, and of these, NfL is the most abundant and soluble subunit, making it the easiest to analyze (Yuan and Rao, 2017). As a consequence of the neuronal damage and loss in the CNS in neurodegenerative diseases, NfL is released into the extracellular spaces and into the CSF and blood, depending on the degree of neuronal damage (Khalil et al., 2018). Several studies have investigated NfL in CSF to identify and monitor neuronal damage and have shown that NfL levels increase in response to neuronal damage, making it a promising biomarker for differentiating neurodegeneration from healthy aging (Zetterberg et al., 2016; Fortea et al., 2018; Rojas et al., 2018; Bridel et al., 2019). Blood NfL is robustly increased in AD (Mattsson et al., 2017; Lin et al., 2018; Ashton et al., 2019b; Preische et al., 2019) but is also increased in many other neurodegenerative disorders and acute neurological disorders (Hansson et al., 2017; Ehler et al., 2019; Hendricks et al., 2019; Mattsson et al., 2019; Ashton et al., 2020; Kapoor et al., 2020; Wihersaari et al., 2021).

However, although NfL in CSF and plasma is found to be strongly increased in neurodegenerative disorders, and other studies point toward saliva being a valid alternative to current methods (Ashton et al., 2019a; Gleerup et al., 2019), salivary NfL has not yet been investigated. Furthermore, several studies have pointed toward the robust stability of NfL in biofluids, and this would be an advantage over biomarkers (e.g., p-tau and amyloid-β) when investigated in an unknown biological matrix with variable collection procedures (Simrén et al., 2021). In this study with consecutive patient inclusion, the levels of NfL in saliva and plasma were investigated to evaluate the diagnostic potential of salivary NfL in a mixed memory clinic cohort.

## Methods

Saliva and plasma samples were collected from all consecutive patients referred for cognitive assessment and lumbar puncture between March 2019 and December 2019 at the Copenhagen Memory Clinic, Copenhagen University Hospital, Rigshospitalet and at the Regional Dementia Research Center, Zealand University Hospital, Roskilde. All samples were analyzed at the Department of Psychiatry and Neurochemistry, the Sahlgrenska Academy, University of Gothenburg. Included patients gave informed consent to participation, and the study was approved by the Danish Data Protection Agency (VD-2019-105) and the Ethical Committee of the Capital Region of Denmark (H-19000651).

### Subjects

A total of 152 patients and 17 healthy controls (HCs) with saliva samples were included in the study. Of these, 135 patients had a matching plasma sample. Saliva samples were collected from HC (*n* = 17), mild cognitive impairment (MCI) (*n* = 47), AD (*n* = 49), and non-AD (*n* = 56) patients. The non-AD group consisted of patients diagnosed with vascular dementia (VaD) (*n* = 10), mixed dementia (*n* = 7), frontotemporal dementia (FTD) (*n* = 9), dementia with Lewy bodies (DLB) (*n* = 6), normal pressure hydrocephalus (NPH) (*n* = 10), alcohol-induced dementia (*n* = 5), and other dementias of unknown etiology (*n* = 5) or dementia due to other neurological or non-neurodegenerative diseases (*n* = 4). The included patients were all diagnosed at an interdisciplinary consensus conference after clinical evaluation, including structural imaging (magnetic resonance imaging or computerized tomography) and, in most instances, 18F-flourdeoxyglucose positron emission tomography (18F-FDG-PET). Patients underwent neuropsychological examination, and furthermore, CSF Aβ_42_, CSF p-tau, and CSF t-tau were included in the diagnostic process with a cutoff of Aβ_42_ of 875 pg/ml (Tijms et al., 2018). The HC participants did not fulfill any of the criteria for neither dementia nor MCI, and the HCs were recruited for research purposes only. All included HCs were Aβ negative. All included patients diagnosed with MCI fulfilled the criteria suggested by the International Working Group in Mild Cognitive Impairment (Winblad et al., 2004), while the patients with AD fulfilled the National Institute on Aging and Alzheimer’s Association (NIA-AA) criteria (McKhann et al., 2011). For the non-AD group, patients with VaD fulfilled the International Society for Vascular Behavioral and Cognitive Disorders (VASCOG) criteria (Sachdev et al., 2014), and patients diagnosed with mixed dementia fulfilled both the NIA-AA criteria and the VASCOG criteria (McKhann et al., 2011; Sachdev et al., 2014). The patients with FTD fulfilled the criteria for behavioral variant (Rascovsky et al., 2011), non-fluent aphasia (Gorno-Tempini et al., 2011), or semantic variant (Gorno-Tempini et al., 2011). Patients with DLB fulfilled the criteria from the fourth report of the DLB consortium (McKeith et al., 2017), patients with NPH were diagnosed according to international guideline criteria for idiopathic normal pressure hydrocephalus (iNPH) (Relktin et al., 2005), while the diagnosis alcohol-induced dementia was established according to the International Classification of Diseases (ICD)-10 criteria (ICD-10,2020).

### Sample

#### Saliva and Plasma Collection

At the Copenhagen Memory Clinic, Copenhagen University Hospital, Rigshospitalet, saliva samples were collected around noon, while saliva samples from the Regional Research Center, Zealand University Hospital, were collected between 9:15 and 10:15 AM. All subjects provided a 1–3-ml whole unstimulated saliva sample in a 15-ml polypropylene falcon tube. Prior to sampling, all participating subjects were asked to abstain from drinking, eating, and smoking, and furthermore requested to drink and swallow some water to rinse their mouth. Whole blood was collected immediately prior to saliva sampling in ethylenediaminetetraacetic acid (EDTA)-treated tubes.

#### Sample Processing

All saliva samples were placed on ice immediately after sampling until centrifugation. Saliva and blood samples were centrifuged at 2,000 *g*, 4°C for 10 min, and redistributed in 250-μl aliquots, avoiding the debris pellet for saliva samples. Saliva and plasma were stored at −80°C until further analysis.

#### Biomarker Assays

Saliva and plasma NfL concentrations were measured using Single molecule array (Simoa) technology and the NF-light Advantage kit (Quanterix, Billerica, MA, United States). Plasma samples were diluted fourfold, and the assay was performed according to instructions from the kit manufacturer. Saliva samples were centrifuged at 10,000 *g*, diluted twofold and analyzed in singlicate. All samples were analyzed in one round of experiments, and the intra-assay coefficients of variation were <10% for plasma and <20% for saliva, as determined by quality control samples analyzed in duplicate. Saliva was analyzed for levels of total protein using the Pierce BCA Protein Assay Kit (Thermo Fisher Scientific). As part of the clinical routine, CSF was analyzed for levels of Aβ_42_, total tau, and phospho-tau using INNOTEST enzyme-linked immunosorbent assays (Fujirebio, Ghent, Belgium).

### Statistical Analyses

The statistical analyses were performed using GraphPad Prism. To test for normal distribution, an Anderson–Darling test was performed. All data on NfL in saliva and plasma and total protein in saliva followed a non-normal distribution and were logarithmic transformed. After logarithmic transformation, the data still did not follow a normal distribution, and therefore, all data were analyzed using a non-parametric test. To analyze NfL levels in saliva and plasma for HC, MCI, AD, and non-AD, a Kruskal–Wallis test were performed. Furthermore, salivary NfL was normalized to the levels of salivary total protein due to considerable variations in salivary total protein. To assess the normalized levels of saliva NfL concentration, a Kruskal–Wallis test was performed. As a sub-analysis, NfL in saliva and plasma and salivary total protein were investigated by a Kruskal–Wallis test between the different diagnoses in the non-AD group. The association between normalized, salivary NfL and NfL in plasma was assessed by Spearman’s rank-order correlation coefficient. Furthermore, Spearman’s rank-order correlation coefficients were used to investigate a potential relationship between the levels of Aβ_42_, p-tau, and tau in CSF and the levels of NfL in saliva. Statistical significance for all analyses was set at *P* < 0.05, two-sided.

## Results

### Demographics

A total of 152 patients and 17 HCs with saliva samples were included in the study, and 135 of these had a matching plasma sample. Table 1 describes the demographic characteristics of the cohort, and Supplementary Table 5 gives more details on the demographic characteristics of the non-AD group. Significant differences were found between the sex distribution, Mini-Mental State Examination (MMSE), and the levels of Aβ_42_, p-tau, and total tau in the CSF as expected. No significant difference was observed on age between the four groups. For the non-AD group (Supplementary Table 4), significant differences were found between the sex distribution, age, and the CSF levels of Aβ_42_ and total tau. No significant differences were observed for MMSE and the CSF levels of p-tau.

**TABLE 1:** Characteristics of the study cohort.

	HC (*n* = 17)	MCI (*n* = 47)	AD (*n* = 49)	Non-AD (*n* = 56)	*P*-value
**Sex F/M**	6/11	22/25	29/20	21/35	0.016*
**Age, years †**	68.4 ± 8.3	71.1 ± 8.2	72.7 ± 7.5	74.0 ± 8.9	0.068
**MMSE score †**	28.9 ± 0.8	26.8 ± 3.2	22.9 ± 4.3	22.5 ± 4.2	<0.0001
**CSF A***b***_42_ (pg/ml) †**	1,062.2 ± 218.2	876.3 ± 244.9	668.0 ± 179.1	901.2 ± 312.4	<0.0001
**CSF p-tau (pg/ml) †**	56.3 ± 20.4	52.2 ± 22.6	92.1 ± 51.2	55.0 ± 29.8	0.002
**CSF total tau (pg/ml) †**	256.7 ± 141.3	343.1 ± 196.4	582.6 ± 241.3	336.0 ± 194.3	<0.0001

*n*, number; F, female; M, male; MMSE, Mini-Mental State Examination; CSF, cerebrospinal fluid; Aβ_42_, amyloid 1-42; p-tau, phosphorylated tau; HC, healthy control; MCI, mild cognitive impairment; AD, Alzheimer’s disease. ^†^Expressed as mean ± standard deviation (SD). *P*-values were calculated by a one-way ANOVA, except *, which was calculated by a Chi-square test.

### Assay Validation for Salivary Neurofilament Light Chain

The commercially available NF-light assay was assessed for its suitability in saliva. The Supplementary Methods and Results detail a partial validation of the assay for saliva analysis. The repeatability measurements for NfL in 12 saliva samples was 20% (Supplementary Table 2 and Supplementary Figure 1) with a high correlation between the repeated samples (*r* = 0.90, *P* < 0.001, Supplementary Figure 1). The linear dilution of four saliva samples demonstrated an average recovery of 97.3% but demonstrated a large range of recovery results (66.6–124%) across all samples but was with range for twofold dilution, which was utilized in this study (Supplementary Table 3). The average spike recovery of NfL calibrator in saliva was on average 63.3% (Supplementary Table 4).

### Neurofilament Light Chain Levels in Saliva and Plasma

Table 2 provides an overview of the mean concentrations of NfL in saliva and plasma, the concentration of total protein in saliva, and the normalized salivary NfL levels.

**TABLE 2:** Mean levels of the biomarkers in saliva and plasma.

	HC (*n* = 17)	MCI (*n* = 47)	AD (*n* = 49)	Non-AD (*n* = 56)	*P*-value*
Saliva NfL (pg/ml)^†^	2.3 ± 2.0	1.8 ± 1.4	2.1 ± 1.6	2.1 ± 1.7	0.79
Plasma NfL (pg/ml)^†^	12.5 ± 4.3	20.6 ± 10.5	24.0 ± 14.3	31.8 ± 24.7	<0.0001
Saliva total protein (μg/ml)^†^	919.6 ± 382.5	953.2 ± 423.0	948.9 ± 461.3	917.4 ± 388.4	0.99
Saliva NfL/saliva total protein^†^	4.4 ± 5.8	2.8 ± 3.3	3.2 ± 5.2	2.8 ± 2.8	0.88

NfL, neurofilament light chain; HC, healthy control; MCI, mild cognitive impairment; AD, Alzheimer’s disease. ^†^Expressed as mean ± standard deviation (SD). *Analyzed by a Kruskal–Wallis test.

**FIGURE 1:**
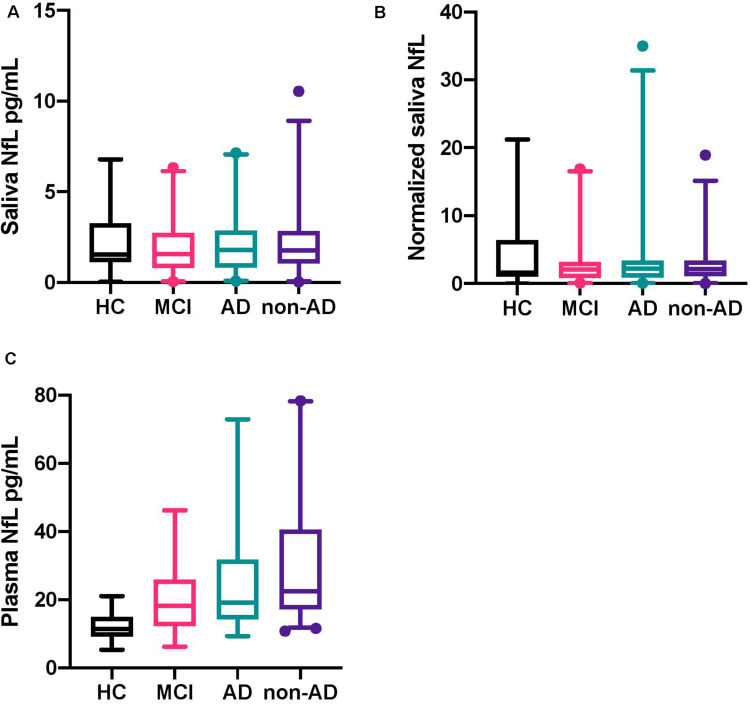
Box plots of neurofilament light chain levels in saliva and plasma and the normalized levels of saliva NfL. **(A)** The boxplots show the median, interquartile range, and the extreme values of salivary NfL for HC, MCI, AD, and non-AD. The 2.5–97.5 percentile of all data had been included in the boxplots. **(B)** The boxplots show the median, interquartile range, and the extreme values of the normalized levels of salivary NfL for HC, MCI, AD, and non-AD. The 2.5–97.5 percentile of all data had been included in the boxplots. **(C)** The boxplots show the median, interquartile range, and the extreme values of plasma NfL for HC, MCI, AD, and non-AD. The 2.5–97.5 percentile of all data had been included in the boxplots. NfL, neurofilament light chain; HC, healthy controls; MCI, mild cognitive impairment; AD, Alzheimer’s disease.

**FIGURE 2:**
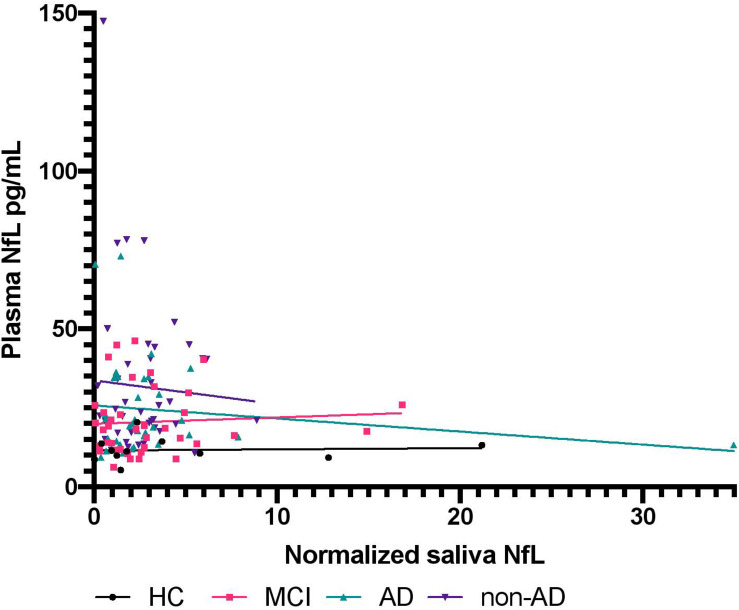
The association between neurofilament light chain in plasma and normalized salivary neurofilament light chain. The figure shows the relationship between NfL in plasma and normalized NfL in saliva for HC, MCI, AD, and non-AD. NIL, neurofilament light chain; HC, healthy controls; MCI, mild cognitive impairment; AD, Alzheimer’s disease.

No statistically significant difference was found between HC, MCI, AD, and non-AD for salivary NfL (*P* = 0.79; Figure 1A). As expected, a statistically significant difference was found for NfL in plasma between the groups (*P* < 0.0001; Figure 1C). When investigating salivary total protein, no significant difference was found (*P* = 0.99), as well as no significant difference for normalized salivary NfL between the diagnostic groups (*P* = 0.88; Figure 1B). For the diagnostic groups contained in the non-AD group, no statistically significant differences were found on the levels of salivary NfL (*P* = 0.57), normalized saliva NfL (*P* = 0.84), and NfL in plasma (*P* = 0.09), respectively. Multiple comparisons tests for Figure 1 can be seen in Supplementary Table 6. Associations, specific to the four groups, between normalized salivary NfL and plasma NfL were investigated, but no statistically significant correlations were found between any of the groups (Figure 2) HC (*P* = 0.86, *r* = 0.20), MCI (*P* = 0.69, *r* = 0.09), AD (*P* = 0.36, *r* = −0.008), and non-AD (*P* = 0.72, *r* = 0.14).

The correlation plots for CSF Aβ_42_, p-tau, and tau and normalized salivary NfL and plasma NfL can be seen in Supplementary Figure 2. No correlations between normalized salivary NfL and Aβ_42_ (*P* = 0.38, *r* = −0.07), p-tau (*P* = 0.37, *r* = 0.07), and tau (*P* = 0.75, *r* = 0.03) were found in the whole dataset. Furthermore, no correlation between plasma NfL and Aβ_42_ (*P* = 0.63, *r* = 0.05) was found in the whole dataset. Statistically significant negative correlations were found between plasma NfL and p-tau (*P* = 0.03, *r* = −0.19) and tau (*P* = 0.04, *r* = −0.19) in the whole dataset. When looking at the individual groups, no correlations were found between normalized salivary NfL and Aβ_42_ (*P* = 0.67, *r* = −0.06), p-tau (*P* = 0.19, *r* = 0.2), and tau (*P* = 0.13, *r* = 0.24) for patients with AD. When investigating MCI, no correlations were found: Aβ_42_ (*P* = 0.18, *r* = −0.20), p-tau (*P* = 0.08, *r* = −0.26), and tau (*P* = 0.07, *r* = −0.28). For the patients with a non-AD dementia diagnosis, no correlations were found between salivary NfL levels and Aβ_42_ (*P* = 0.96, *r* = −0.007), p-tau (*P* = 0.19, *r* = 0.19), and tau (*P* = 0.40, *r* = 0.12). For the HCs, no correlations were found between salivary NfL and Aβ_42_ (*P* = 0.06, *r* = 0.83) and tau (*P* = 0.06, *r* = 0.83). For p-tau, a statistically significant correlation was found (*P* = 0.003, *r* = 1.0). The correlations between plasma NfL levels and Aβ_42_, p-tau, and tau were also investigated in the individual groups. No statistically significant correlations were found between plasma NfL levels and Aβ_42_ (*P* = 0.31, *r* = 0.17; *P* = 0.45, *r* = 0.13; *P* = 0.64, *r* = 0.07; *P* > 0.99, *r* = 0.00), p-tau (*P* = 0.07, *r* = 0.30; *P* = 0.10, *r* = −0.27; *P* = 0.47, *r* = −0.11; *P* > 0.99, *r* = 0.00), and tau (*P* = 0.40, *r* = −0.14; *P* = 0.19, *r* = −0.22; *P* = 0.052, *r* = −0.30; *P* > 0.99, *r* = 0.00) for AD, MCI, non-AD, and HC respectively.

## Discussion

The salivary concentrations of NfL were found to have no significant differences between HC, MCI, AD, and non-AD diagnostic groups. In addition, there was no association of saliva NfL concentration with plasma NfL, CSF Aβ_42_, CSF p-tau, or CSF tau. Plasma NfL, on the other hand, showed the expected statistically significant differences between the diagnostic groups, but these measures did not correlate with saliva NfL.

Comparing NfL concentrations in saliva and plasma, plasma NfL levels were measured to be approximately 10 times higher than in saliva. When comparing NfL in plasma and CSF, studies have shown that the concentration of NfL in CSF is around 100 times higher than in plasma (Olsson et al., 2016). Several studies have suggested that plasma NfL is a promising biomarker for differentiating neurodegeneration from healthy aging (Vågberg et al., 2015; Mielke et al., 2019; Khalil et al., 2020). Our results suggest that this is not replicable using saliva as the biomarker matrix. Some limitations on optimal assay performance should be highlighted; firstly, the average Coefficient of variation (CV) between repeated salivary NfL measurements is substantially greater than what is observed for plasma NfL, which could be due to viscosity in some samples. Secondly, the spike recovery performance is marginally below standard criteria but within criteria for a clinically validated immunoassay (Vanderstichele et al., 2000). However, these limitations cannot account for the lack of association between salivary NfL and neurodegeneration but point toward the need for improved and standardized saliva collection and samples handling for optimal biofluid analysis.

From our data, it is clear that NfL is present in saliva. The lack of a correlation between saliva and plasma NfL concentrations speak against passive leakage from blood. It is possible that NfL is released from nerves innervating the salivary gland or that local production of NfL occurs in the gland. In any case, our results suggest that NfL concentration in saliva does not reflect CNS neurodegenerative disease.

## Conclusion

This was the first study to measure levels of salivary NfL in a consecutive cohort of patients with neurodegenerative dementias. We conclude that saliva NfL concentration can be robustly measured but that the levels do not reflect neurodegeneration within the CNS. In contrast, plasma NfL concentration from the same patients showed the expected group differences. Saliva NfL is not a reliable biomarker for neuronal injury in neurodegenerative disease. Currently, we have no explanation for the appearance of NfL in saliva: is it an artifact of passive leakage from blood, a peripheral nerve expression, or a pathophysiological process? A key question would be to observe salivary NfL in acute disorders where a dramatic increase of blood NfL is observed after the first few days of injury. Furthermore, the fact that NfL can be measured in saliva warrants more studies of reported biomarkers for AD. Saliva is an easily obtained source of biomarkers, and therefore, more studies should investigate saliva in order to understand optimal collection methods and handling and to be ultimately investigated further as a biomarker source for neurodegenerative dementias.

## Data Availability Statement

The raw data supporting the conclusions of this article will be made available by the authors, without undue reservation.

## Ethics Statement

The studies involving human participants were reviewed and approved by the Ethical Committee of the Capital Region of Denmark (H-19000651). The patients/participants provided their written informed consent to participate in this study.

## Author Contributions

HG, SH, and AS contributed to the conceptualization. HG contributed to the data curation, formal analysis, project administration, and writing the original draft. HG, SH, AS, KB, and HZ contributed to the funding acquisition. HG, FS, JS, PH, and NA contributed to the methodology. SH, HZ, NA, and AS contributed to the supervision. NA and AS contributed to the validation. HG, FS, PH, JS, KB, HZ, NA, and AS contributed to the writing, review, and editing. All authors contributed to the article and approved the submitted version.

## Conflict of Interest

KB has served as a consultant, at advisory boards, or at data monitoring committees for Abcam, Axon, Biogen, JOMDD/Shimadzu, Julius Clinical, Lilly, MagQu, Novartis, Roche Diagnostics, and Siemens Healthineers and is a co-founder of Brain Biomarker Solutions in Gothenburg AB (BBS), which is a part of the GU Ventures Incubator Program. HZ has served at scientific advisory boards for Denali, Roche Diagnostics, Wave, Samumed, Siemens Healthineers, Pinteon Therapeutics, Nervgen, and CogRx, has given lectures in symposia sponsored by Fujirebio, Alzecure, and Biogen, and is a co-founder of Brain Biomarker Solutions in Gothenburg AB (BBS), which is a part of the GU Ventures Incubator Program. The remaining authors declare that the research was conducted in the absence of any commercial or financial relationships that could be construed as a potential conflict of interest.
