# Supplementary material for: Saliva Neurofilament Light Chain Is Not a Diagnostic Biomarker for Neurodegeneration in a Mixed Memory Clinic Population
Source: Front Aging Neurosci. 2021 May 10;13:659898. doi: 10.3389/fnagi.2021.659898 (PMC8141589; doi:10.3389/fnagi.2021.659898)
Supplement: Supplementary file 1 [file Data_Sheet_1.pdf]

Supplementary Data

CONTENTS

1 SUPPLEMENTARY METHODS

Analytical verification of saliva NfL by single molecule array (Simoa)

2 SUPPLEMENTARY TABLES AND FIGURES

Supplementary Table 1. Quality control performance

Supplementary Table 2 and Figure 1. Repeatability of salivary NfL

Supplementary Table 3. Dilution linearity of salivary NfL

Supplementary Table 4. Spike recovery performance of the salivary NfL

Supplementary Table 5. Characteristics of the non-AD group

Supplementary Table 6. Multiple comparisons for saliva and plasma NfL and normalized saliva NfL

Supplementary Figure 2. Correlation plots for CSF Aβ42, p-tau and tau, and normalized salivary NfL and plasma NfL

1 SUPPLEMENTARY METHODS

Analytical verification of saliva NfL by single molecule array (Simoa)

The assay verification focused on repeatability dilution linearity and spike recovery. For dilution linearity, saliva samples were analyzed undiluted or diluted (2-fold and 4-fold).

The % recovery was calculated using the following equation:

% recovery at dilution x = (Concentration at dilution x) / (Concentration of undiluted sample) ×100

For spike recovery, neat saliva samples as well as the assay diluent only were each analyzed untreated (non-spiked) or 'spiked' with low or high concentrations of recombinant NfL (assay calibrator)

The following equation was used to calculate spike recovery:

% recovery = (Concentration of spiked sample) / (Concentration of non-spiked + concentration of spiked buffer) ×100

2 SUPPLEMENTARY TABLES AND FIGURES

Supplementary Table 1. Quality control performance

Analyte	Matrix	QC mean (pg/ml)	Intra-assay variability (%)
NfL	Plasma	6.2	6.9
		112.3	4.1
	Saliva	1.7	17.7
		3.3	12.7

Supplementary Table 2 and Figure 1. Repeatability of salivary NfL

A total of 12 saliva samples that were included in the study were once again measured at a later date. The repeatability value for salivary NfL 20.1%.

Supplementary Table 2:

Anonymized sample ID	Original measurement (pg/mL)	Repeated measurement (pg/mL)	CV (%)
001	0.7	1.02	26.6
002	1.29	1.19	5.5
003	0.29	0.49	35.5
004	1.77	1.49	11.8
005	1.07	1.89	39.1
006	1.83	1.94	4.1
007	2.79	3.05	6.4
008	3.13	3.87	15.0

009	1.6	2.61	33.7
010	2.1	3.67	38.9
011	2.8	3.02	5.4
012	2.17	2.79	17.8

Supplementary Figure 1:

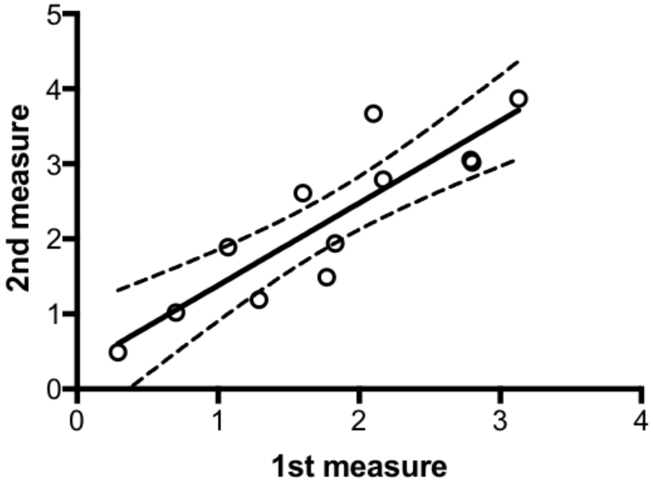

Supplementary Table 3. Dilution linearity of salivary NfL.

Sample No.	Fold Dilution	Calc. conc. (pg/mL)	Dilution corrected conc. (pg/mL)	% Recovery
**1**	1	5.63	5.63	
	2	3.21	6.42	114%
	4	1.75	7	124%
**2**	1	2.66	2.66	

	2	1.12	2.24	84.2%
	4	0.44	1.76	66.6%
**3**	1	3.22	3.22	
	2	1.33	2.66	82.6%
	4	0.76	3.04	94.4%
**4**	1	1.25	1.25	
	2	0.55	1.1	88.8%
	4	0.41	1.64	123.7%

Supplementary Table 4. Spike recovery performance for salivary NfL

Sample	Treatment	Mean observed conc. (pg/ml)	%CV conc.	Expected conc. (pg/ml)	% recovery
1	Neat	4.3	10.9		
	+ high spike	45.1	7.1	79.3	56.9
	+ low spike	17.1	1.5	22.3	76.7
2	Neat	4.2	10.1		
	+ high spike	91.5	5.4	125.2	73.1
	+ low spike	7.8	2.3	9.8	79.6
3	Neat	1.9	14.3		

	+ high spike	34.2	3.6	48.2	70.9
	+ low spike	5.2	8.9	7.1	73.2
**Buffer control**	Buffer + high spike	73.4	1.55	75	99.2
	Buffer + low spike	9.25	7.9	8	115

Supplementary Table 5. Characteristics of the non-AD group

**Supplementary table 5**	**VaD (n=10)**	**Mixed (n=7)**	**FTD (n=9)**	**DLB (n=6)**	**NPH (n=10)**	**Alcohol induced dementia (n=5)**	**Dementia of unknown etiology (n=5)**	**Dementia due to neurological or non-neurodegenerative diseases (n=4)**	**p-value**
**Sex F/M**	4/6	4/3	4/5	2/4	2/8	1/4	3/2	1/3	0.03*
**Age, years †**	79.1 ± 5.5	75.6 ± 9.0	75.9 ± 3.1	73.3 ± 7.0	76.0 ± 7.6	69.0 ± 7.4	71.6 ± 7.2	59.5 ± 13.7	0.01
**MMSE score †**	21.3 ± 3.9	22.2 ± 2.7	24.1 ± 4.0	22.6 ± 5.0	22.2 ± 4.3	22.2 ± 3.3	22.4 ± 3.7	23.3 ± 6.6	0.95
**CSF Ab_{42} (pg/mL) †**	890.0 ± 290.5	619.9 ± 191.9	1032.3 ± 272.2	791.0 ± 323.1	779.1 ± 192.9	1178.4 ± 80.0	1046.0 ± 286.7	1168.7 ± 415.1	0.01
**CSF p-tau (pg/mL) †**	46.7 ± 16.6	85.4 ± 33.5	60.2 ± 28.9	51.3 ± 17.3	43.9 ± 22.9	39.2 ± 15.7	48.0 ± 16.2	69.8 ± 50.8	0.08
**CSF total tau (pg/mL) †**	250.6 ± 58.1	653.6 ± 305.6	326.0 ± 64.7	348.3 ± 128.8	224.8 ± 101.8	248.8 ± 82.1	290.8 ± 117.6	293.7 ± 139.5	<0.0001

Abbreviations: n, number; F, female; M, male; MMSE, mini mental state examination; CSF, cerebrospinal fluid, $A\beta_{42}$; amyloid 1-42; p-tau, phosphorylated tau; HC, healthy controls; MCI, mild cognitive impairment; AD, Alzheimer’s disease.

The non-AD group (n=56) consisted of patients diagnosed with vascular dementia (VaD) (n=10), mixed dementia (n=7), frontotemporal dementia (FTD) (n=9), dementia with Lewy bodies (DLB) (n=6), normal pressure hydrocephalus (NPH) (n=10), alcohol-induced dementia (n=5) and other

dementias of unknown etiology (n=5) or dementia due to other neurological or non-neurodegenerative diseases (n=4). † are expressed as mean ± standard deviation (SD). *P-values were calculated by a one-way ANOVA, except **, which was calculated by a Chi-squared test.

Supplementary Table 6. Multiple comparisons for saliva and plasma NfL and normalized saliva NfL

**Supplementary Table 6A. Saliva NfL**	
**Dunn's multiple comparisons test**	**p-value**
HC vs. MCI	>0.99
HC vs. AD	>0.99
HC vs. non-AD	>0.99
MCI vs. AD	>0.99
MCI vs. non-AD	>0.99
AD vs. non-AD	>0.99

**Supplementary Table 6B. Normalized Saliva NfL**	
**Dunn's multiple comparisons test**	**p-value**
HC vs. MCI	>0.99
HC vs. AD	>0.99
HC vs. non-AD	>0.99
MCI vs. AD	>0.99
MCI vs. non-AD	>0.99
AD vs. non-AD	>0.99

**Supplementary Table 6C. Plasma NfL**	
**Dunn's multiple comparisons test**	**p-value**
HC vs. MCI	0.04
HC vs. AD	0.003
HC vs. non-AD	<0.001
MCI vs. AD	>0.99
MCI vs. non-AD	0.055
AD vs. non-AD	0.74

Abbreviations: HC, healthy controls; MCI, mild cognitive impairment; AD, Alzheimer's disease.

Supplementary Fig. 6A): The table shows Dunn's multiple comparisons test for salivary NfL. 6B): The table shows Dunn's multiple comparisons test for normalized salivary NfL. 6C): The table shows Dunn's multiple comparisons test for plasma NfL.

Supplementary Figure 2. Correlation plots for CSF Ab42, p-tau and tau, and normalized salivary NfL and plasma NfL

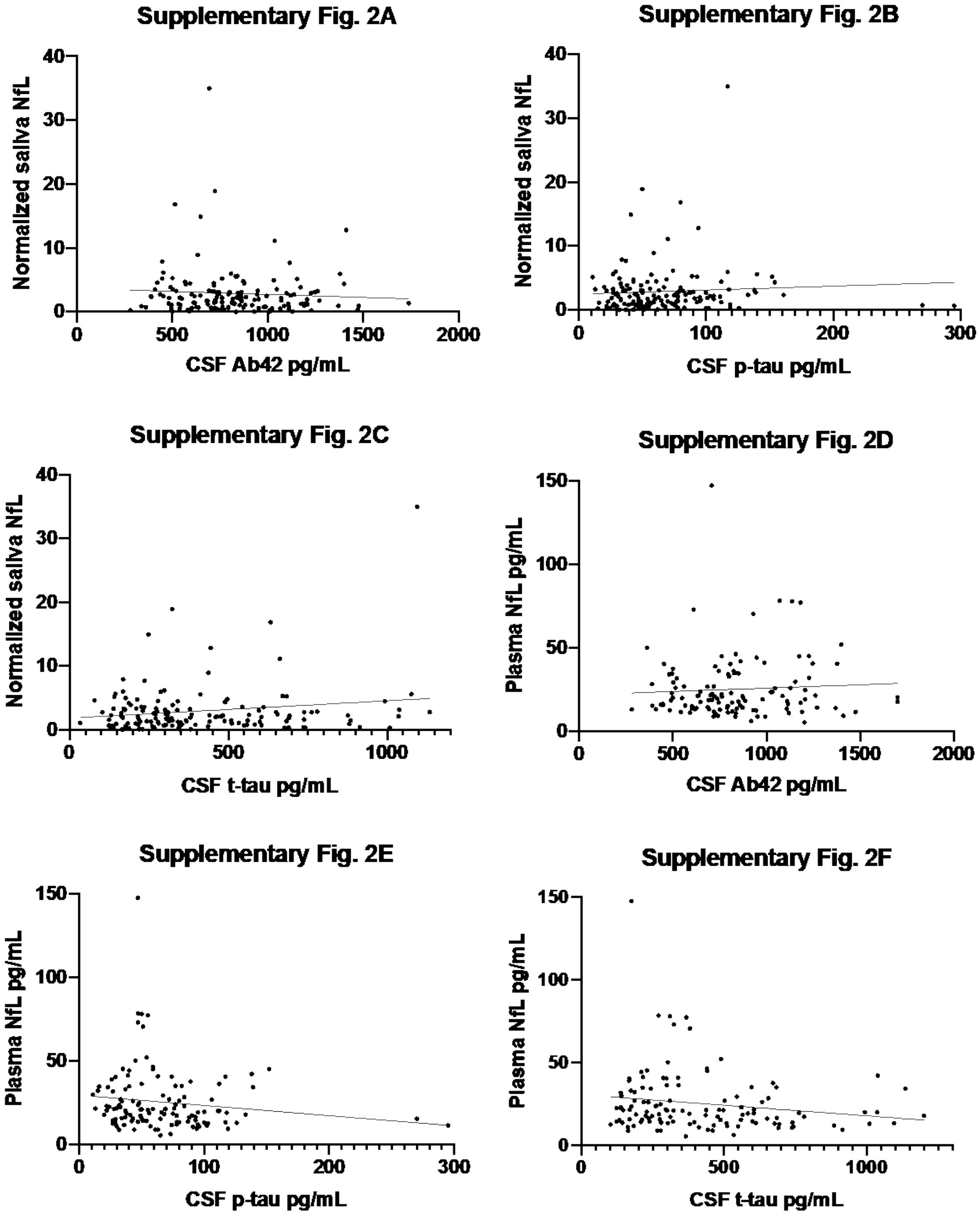

Supplementary Fig. 2A: The graph shows the correlation between Aβ42 in CSF and normalized salivary NfL. Supplementary Fig. 2B: The graph shows the correlation between p-tau in CSF and normalized salivary NfL. Supplementary Fig. 2C: The graph shows the correlation between t-tau in CSF and normalized salivary NfL. Supplementary Fig. 2D: The graph shows the correlation between Aβ42 in CSF and plasma NfL. Supplementary Fig. 2E: The graph shows the correlation between p-tau in CSF and plasma NfL. Supplementary Fig. 2F: The graph shows the correlation between t-tau in CSF and plasma NfL.

Abbreviations: NfL, neurofilament light chain; Aβ42, beta amyloid 1-42; p-tau, phosphorylated tau; t-tau, total tau; CSF, cerebrospinal fluid
